# A Ranking System for Reference Libraries of DNA Barcodes: Application to Marine Fish Species from Portugal

**DOI:** 10.1371/journal.pone.0035858

**Published:** 2012-04-25

**Authors:** Filipe O. Costa, Monica Landi, Rogelia Martins, Maria H. Costa, Maria E. Costa, Miguel Carneiro, Maria J. Alves, Dirk Steinke, Gary R. Carvalho

**Affiliations:** 1 CBMA (Centre of Molecular and Environmental Biology), Department of Biology, University of Minho, Campus de Gualtar, Braga, Portugal; 2 INRB, IP/IPIMAR, Lisboa, Portugal; 3 IMAR – Instituto do Mar, Departamento de Ciências e Engenharia do Ambiente, Faculdade de Ciências e Tecnologia, Universidade Nova de Lisboa, Caparica, Portugal; 4 Centro de Ciências do Mar (CCMAR), Universidade do Algarve, Faculdade de Ciências e Tecnologias (FCT), Campus de Gambelas, Faro, Portugal; 5 Museu Nacional de História Natural & Centro de Biologia Ambiental, Universidade de Lisboa, Lisboa, Portugal; 6 Biodiversity Institute of Ontario University of Guelph, Guelph, Ontario, Canada; 7 Molecular Ecology and Fisheries Genetics Laboratory, School of Biological Sciences, Bangor University, Bangor, Gwynedd, Wales; American Museum of Natural History, United States of America

## Abstract

**Background:**

The increasing availability of reference libraries of DNA barcodes (RLDB) offers the opportunity to the screen the level of consistency in DNA barcode data among libraries, in order to detect possible disagreements generated from taxonomic uncertainty or operational shortcomings. We propose a ranking system to attribute a confidence level to species identifications associated with DNA barcode records from a RLDB. Here we apply the proposed ranking system to a newly generated RLDB for marine fish of Portugal.

**Methodology/Principal Findings:**

Specimens (n = 659) representing 102 marine fish species were collected along the continental shelf of Portugal, morphologically identified and archived in a museum collection. Samples were sequenced at the barcode region of the cytochrome oxidase subunit I gene (COI-5P). Resultant DNA barcodes had average intra-specific and inter-specific Kimura-2-parameter distances (0.32% and 8.84%, respectively) within the range usually observed for marine fishes. All specimens were ranked in five different levels (A–E), according to the reliability of the match between their species identification and the respective diagnostic DNA barcodes. Grades A to E were attributed upon submission of individual specimen sequences to BOLD-IDS and inspection of the clustering pattern in the NJ tree generated. Overall, our study resulted in 73.5% of unambiguous species IDs (grade A), 7.8% taxonomically congruent barcode clusters within our dataset, but awaiting external confirmation (grade B), and 18.7% of species identifications with lower levels of reliability (grades C/E).

**Conclusion/Significance:**

We highlight the importance of implementing a system to rank barcode records in RLDB, in order to flag taxa in need of taxonomic revision, or reduce ambiguities of discordant data. With increasing DNA barcode records publicly available, this cross-validation system would provide a metric of relative accuracy of barcodes, while enabling the continuous revision and annotation required in taxonomic work.

## Introduction

How many marine fish species are there in Portugal? The answer to this apparently simple question may not be straightforward, at least not at present. The marine ichthyofauna of Portugal is probably among the richest among European countries. FishBase reports 501 marine species for continental Portugal, in addition to 325 and 590 species in the Azores and Madeira archipelagos, respectively [1]. If all species common in these three regions are considered, the total catalogue amounts to 828 species. In fact, the number is probably higher, as all species lists in FishBase are labelled as incomplete. If we consider the whole area covered by the Portuguese Exclusive Economic Zone (EEZ), one of the largest in Europe, the number of fish species is likely to increase even further.

Because of its geographic location and size, Portugal's EEZ can potentially serve as a meeting ground for fish species from many different adjacent sources, such as the Mediterranean Sea, the subtropical Northeastern Atlantic, the depths of the mid-Atlantic ridge, the cold temperate Northeastern Atlantic and possibly as well the temperate Northwestern Atlantic. Portugal's geographic location appears particularly well suited to monitor temporal changes in marine fish species ranges of the Northeastern Atlantic [2]. However, the biologically and zoogeographically diverse and dynamic nature of the ichtyofauna in the region poses particular challenges for fisheries biologists and others that routinely need to identify fish species rapidly.

These researchers are also exposed to the well-reported universal difficulties in fish species identifications, as for example cases of species with limited diagnostic morphological features, cryptic species, juvenile identification, or unavailability of adequate drawings and descriptions [3], [4]. Indeed, a report by Lleonart et al. (2006) [5] indicates that such problems are global, with almost 34% of the world's fisheries catch from 1950–2002 lacking species level identification. Such reduced species-specific catch data has undermined fish stock assessment and sustainability of fisheries at both global and regional levels [5]. Moreover, among the circa 66% of the catch assigned to species, there is no approach to measure how rigorous the identifications were, and no method in place to estimate the percentage of species mis-assignments. Hence, identification errors may accumulate in fisheries statistics unnoticed, eventually leading to gross inaccuracies in estimating species abundances and projected recruitment, with detrimental consequences for conservation and fishery management.

Molecular markers, such as DNA barcodes [6], can address many such difficulties, namely due to their universal applicability and direct comparability among geographic regions, their reduced ambiguity, and the ease of use by non-experts [3]. In addition to the use of DNA barcodes for species delimitation, the availability of a standardized and globally accessible database (Barcode of Life Data System, BOLD, http://www.barcodinglife.org) [7] facilitates numerous related applications, including detection of putative cryptic species, identifications of ambiguous life history stages, estimates of shifts in species ranges, issues relating to traceability, illegal fishing and fish fraud, and the analysis of food webs and trophic dynamics [3].

Here we report the launch of a globally-accessible reference library of DNA barcodes (RLDB) for circa 100 fish species from Portuguese waters. We focused our efforts on species occurring along the continental coast of Portugal, collected both during fisheries surveys and as by-catch in trawling fisheries boats. By using the resulting reference library we tested the discriminatory capability of DNA barcodes and identified major taxonomic misidentification and challenges.

Because taxonomic identifications and published sequences are susceptible to occasional inaccuracies [8], [9], [10], a continuous process of confirmation and validation is required to build a comprehensive and accurate reference library. We therefore propose an empirical ranking system for the current dataset that will provide end-users with a benchmark of reliability associated with each DNA barcode. As the global fish barcode library is increasingly populated, ranks are subject to review and changed accordingly when justified. The resultant fish DNA barcode library is meant to constitute a valuable base-line resource for ichthyologists, fisheries biologists and other professionals that need rigorous and reliable species identifications on a routine basis and often for multi-species catches comprising various life history stages.

## Methods

### Sampling

**Figure 1:**
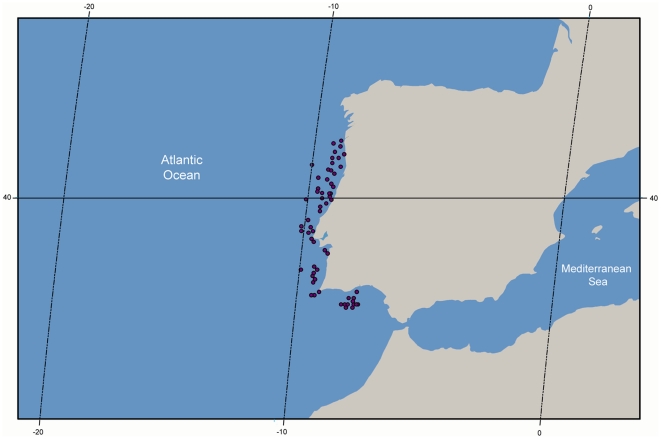
Sampling locations of marine fishes along Portuguese mainland coast. Yellow circle – 1–5 specimens, orange circle – 5–50 specimens, red circle – >50 specimens.

The specimens were collected along the continental coast of Portugal (Fig. 1) during 3 surveys on research vessels of the Instituto Português de Investigação Marítima (IPIMAR), in March and October 2005, and in June 2006. Additional specimens were collected from legal fishing boats operating off the south coast of Portugal during the year 2005, using trawling, seine and trammel nets. Full details of the latter sampled fish species are available elsewhere [11]. Preliminary species identification was attributed to each specimen immediately after collection, which was later verified in the laboratory. Upon identification, muscle tissue samples were taken, and preserved in 96% ethanol for later molecular analyses. All specimens were subsequently registered and archived in the National Museum of Natural History, Lisbon, except poorly preserved or damaged individuals. At least one representative specimen of each species was archived. A total of 659 DNA barcodes were assembled from 102 fish species using the protocol detailed below. Among these, 9 barcode sequences were obtained from tissue samples of the species *Galeus melastomus* and *Galeus atlanticus* obtained from a separate study [12]. DNA barcodes for 33 fish species were generated and are publicly available for the first time. A complete list of species and specimens examined in this study is provided in Table S1, and detailed collection and DNA sequence data for each specimen are available from the Barcode of Life Data Systems (BOLD) website [7] in the project “DNA barcoding Fishes of Portugal”.

### DNA Isolation, Amplification and Sequencing

Two distinct protocols were applied in the molecular analyses, depending on the laboratory where samples were processed, namely at the Biodiversity Institute of Ontario (BIO) or at Bangor University. Both protocols are very similar, differing only in the DNA extraction method and PCR reaction. The protocol used at BIO has been described elsewhere [13]. Here we detail the protocol applied at Bangor University: DNA was extracted from white muscle tissue, by using Chelex Dry Release method [14]. The 652 bp barcode region of the mitochondrial gene cytochrome oxidase I COI (hereafter, COI-5P) was subsequently amplified based on the PCR protocol and cycling conditions proposed for fish by Ivanova et al. [15]. Either the primer cocktails C_VF1LFt1–C_VR1LRt1 or C_FishF1t1–C_FishR1t1 were used in a 25 µL- PCR reaction containing 14 µl of ultrapure water, 5 µl of 5× PCR Buffer, 2.5 µl of MgCl_2_ (25 mM), 0.25 µl of dNTPs (10 mM), 0.5 µl of each forward and reverse primer cocktail (0.01 mM), 0.25 µl of U/µL *Taq* DNA Polymerase (Promega) and 2.0 µl of DNA template. The PCR amplicons were subsequently visualized on a 1% agarose gel and purified with the addition of 10 U of Exonuclease I and 1 U Shrimp Alkaline Phosphate incubated at 37°C for 15 min, followed by 15 min at 80°C. Both forward and reverse DNA strands were sequenced at Macrogen Inc., using an ABI 3730 capillary sequencer. Forward and reverse COI-5P sequences were edited and aligned using MEGA version 4.1 [16], and submitted to BOLD and GenBank (Accessions JQ774505 – JQ775163, Table S1).

### Data Analyses

#### DNA barcodes discriminatory ability

We determined COI-5P average pairwise divergences at different taxonomic levels (within-species, congeneric, and confamilial divergences) using the Kimura 2-parameter (K2P) distance model [17], as implemented in the “distance summary” tool available in BOLD. The software MEGA [16] was used to build a Neighbour-Joining (NJ) tree using the K2P distance model, to examine the aggregation of species into clusters. Bootstrap values for each node were estimated by 1000 replications. We also generated a Klee diagram based on indicator vector correlations for analyzing and displaying affinities of COI-5P haplotypes [18], [19]. Using this method, uncorrected COI-5P haplotype sequences were transformed into digital indicator vectors using M = 2 sequences per species, generating unique representations of each sequence in the chosen vector space [18]. A false-color map depicts correlations among the species indicator vectors. The succession of species for this approach is provided in Table S2.

#### Ranking method for attributing a taxonomic reliability grade to reference DNA barcodes

**Figure 2:**
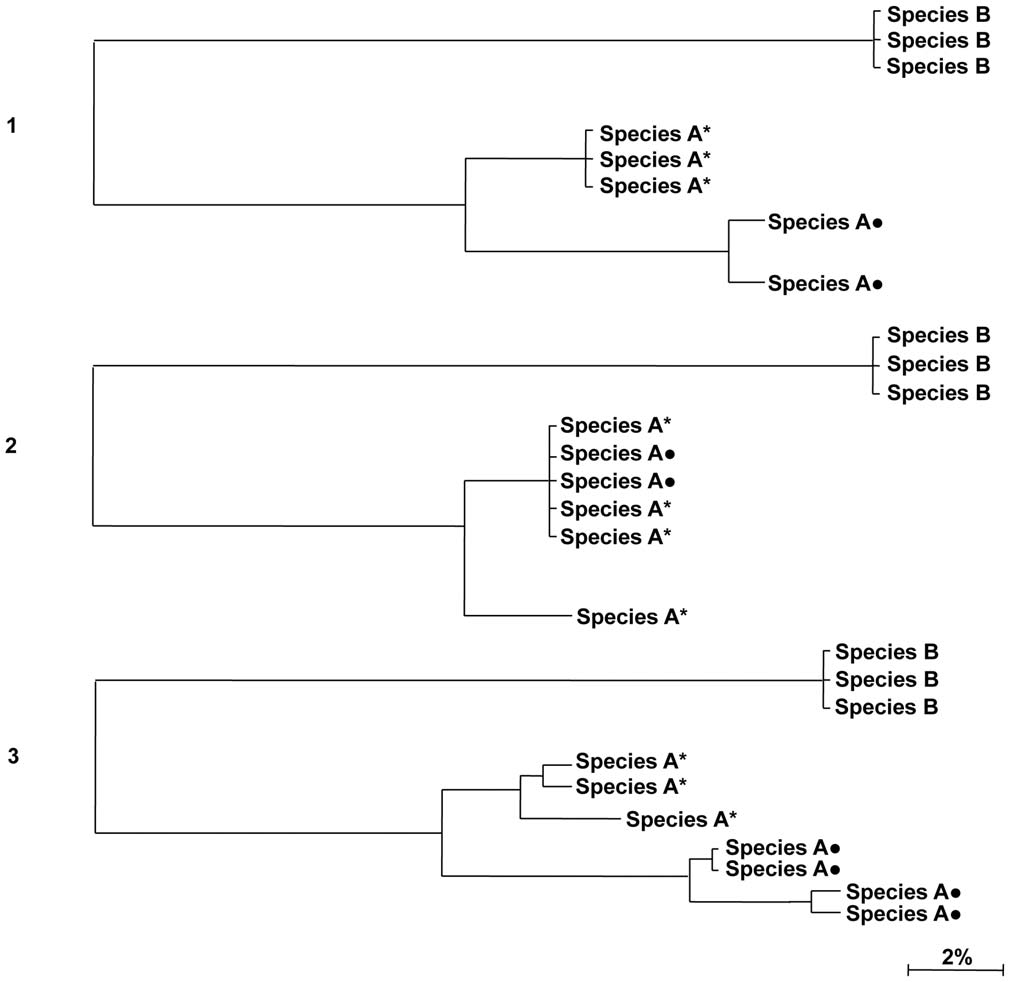
Three possible scenarios for attribution of Grade C for species included in a reference library of DNA barcodes. 1- Specimens available from our library reveal genetic distance greater than 2% compared with conspecifics available from other projects. 2- Conspecific distance greater than 2% is observed within our dataset and across datasets available in BOLD. 3- Intra-specific genetic structure (genetic distance >2%) both within our dataset and in other projects available in BOLD. * Our dataset; **•** External dataset.

We established an empirical ranking system to assess the level of taxonomic reliability of species-specific DNA barcode arrays in our reference library. The underlying rationale of the ranking system is that taxonomic reliability is greater if DNA barcode sequence data from multiple independent observers produce congruent and unambiguous matches for a given species. To check for species ID congruence among independent observers, we submitted a representative COI-5P sequence of each species in our dataset to the BOLD-IDS. A sequence was selected as representative if it was member of the most abundant haplotype, had maximal length and showed no ambiguous base-calls. We used the Species level search option in the BOLD-IDS, and evaluated the probability of placement of our query sequence in a species. The Species level search option represents a subset of BOLD that contains every COI-5P barcode record with a species level identification and a minimum sequence length of 500 bp. This includes many species represented by only one or two specimens as well as all species with interim taxonomy. We also examined the clustering pattern of our sequences in the NJ tree provided by the BOLD-IDS (Tree Based Identification), which places the query sequence within a NJ among 100 of the nearest matching sequences available in BOLD [7]. Based on the results of the query above, an arbitrary measure (from A- highest, to E- lowest reliability) of taxonomic reliability was attributed to each DNA barcode according to the following criteria:

1. External concordance: unambiguous species match with specimens from other BOLD projects or published sequences. Monophyletic species with a maximum of 2% (patristic) sequence divergence [20].
2. Internal concordance: species congruent within our dataset, where at least 3 specimens of the same species are available, with a maximum of 2% (patristic) sequence divergence [20]. No matching sequences found through the BOLD-IDS.
3. Sub-optimal concordance (possible within species genetic structure): at least 3 specimens of the same species are available within the library and form a monophyletic cluster; however intraspecific distance is greater than 2%; and/or the BOLD-IDS indicates monophyletic nearest neighbour of the same species, with more than 2% patristic distance (Fig. 2).
4. Insufficient Data: low number of specimens analysed (1 or 2 individuals) and no matching sequence available in BOLD.
5. Discordant species assignments: sequences for a given species in our dataset did not match with the same species in BOLD. The specimen may match with a different species or may display paraphyly or polyphyly.

For discordant species IDs among DNA barcode clusters, we defined various possible reasons for their occurrence:

1. Morphology-based misidentifications – one or several independent observers misidentified well-established species. This case typically occurs when one or more species of the same genus cluster together.
2. Taxonomic uncertainty – known cases of uncertainty of the taxonomic status of the species involving possible synonyms or also near-cryptic species whose morphological identification is particularly difficult and leads to frequent misidentifications.
3. Barcode sequence sharing – cases of clearly established species that cannot be distinguished by DNA barcode sequences, due to recent divergence or introgressive hybridization of mitochondrial DNA.
4. Flaws in sample processing – consisting in possible errors during the manipulation of specimens, specimen data or molecular analyses, leading to errors such as mislabelling, or cross-contamination of DNA templates. These types of errors are prominent and readily detected, as for example when specimens from different orders or families cluster together. These situations were judged case-by-case and, whenever sequences from other studies appear clearly misplaced within a cluster they were not considered for grade attribution of our specimens.

All specimens in the database were attributed a grade according to the ranking system above, which is displayed in the “extra info” field of the public project lodged in BOLD titled “DNA barcoding Fishes of Portugal”. Along with the respective grade, the date of the attribution and the initials of the researcher are provided for control in potential future updates.

## Results

### Sampling breadth and global divergence patterns

A total of 102 species have been analysed, distributed across 79 genera, 54 families, and 22 orders (Table S1). Seventy species were represented by at least 3 specimens, with an average of 6 individuals per species (range 1–18) for the entire dataset. The DNA barcodes of thirty-three species were obtained for the first time compared to the global library of COI-5P sequences publicly available in GenBank.

**Figure 3:**
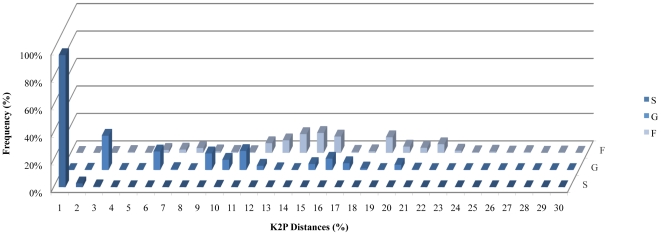
Frequency distibution of COI-5P distances (K2P distance model) for marine fish species from Portugal. A total of 102 species were analysed, distributed in 79 genera and 54 families. Within-species (S), within-genus (G), and within-family (F).

**Figure 4:**
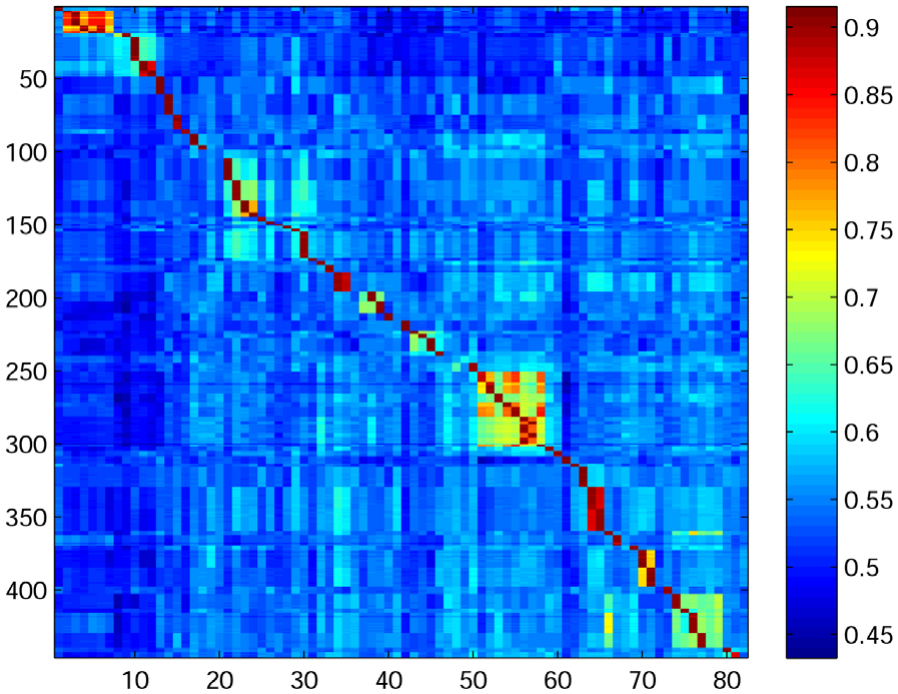
Indicator vector correlations of the COI-5P data set (Klee diagram). The false-color representation depicts correlations among 447 COI-5P test sequences (y-axis) and 83 species-level indicator vectors (x-axis). In total, 430 (96.2%) of the test sequences showed highest correlation with their respective species indicator vector.

Molecular analyses yielded 659 COI-5P barcodes (97% with 600–652 bp; 3% with 430–599 bp), characterized by the absence of stop codons, insertions or deletions. Overall, within-species K2P mean distance was 25× lower than average congeneric distance (0.32% and 8.84%, respectively; Table 1). The average confamilial distance was 15.03%, ranging from 3.12% (Triglidae) to 29.00% (Callionymidae and Serranidae) (Fig. 3). The analysis of the discriminative power of COI-5P revealed that all 102 species were distinguishable within our dataset, with species grouping into distinct, non-overlapping clusters in the NJ tree (Fig. S1). The maximum conspecific divergences were observed for *Scorpaena notata* (18.56%). Maximum congeneric distances were observed for the genus *Microchirus* (20%). The lowest congeneric distance (1.88%) was recorded between *Trachurus trachurus* and *T. picturatus*, although they were arranged in two clearly separated monophyletic clusters with 17 specimens each. This represents the only case of congeneric divergence below 2% in our dataset. The Klee diagram revealed correlation values >0.70 among members of the families Triglidae and Rajidae, indicating a high similarity of the uncorrected COI-5P sequences among these species pairs (Fig. 4). The scorpionfish *Scorpaena notata* displayed intraspecific genetic structure, which is apparent through a clustering pattern of two distinct clades, each supported by 99% confidence (Fig. S1), that diverge on average by 8.94% (K2P). Three families out of 54 were polyphyletic in the NJ tree, namely Lotidae, Gadidae, and Sparidae (Fig. S1).

**Table 1:** Pairwise average COI-5P barcode distances of marine fish species^1^ using Kimura 2-Parameter model.

Comparison Intra-	No. of Comparisons	Minimum Distance	Mean Distance± SE	Maximum Distance
**Species**	3057	0	0.322±0.02	18.555
**Genus**	1404	1.875	8.842±0.137	20.328
**Family**	4099	3.124	15.042±0.069	29.747
**Order**	23828	9.599	23.613±0.021	42.032

1 Values are estimated from 659 COI-5P barcodes of 102 species from Portugal.

### Grades of taxonomic reliability

Application of the ranking system to all analysed 102 fish species resulted in 73.5% of the species taxonomic identifications congruent with external data (BOLD), hence eligible for Grade A in our ranking system. Taxonomically congruent identifications within our dataset only, due to the absence of matching COI-5P sequences in BOLD, were represented in 7.8% of species (Grade B), while the remaining 2.9% of the total were attributed Grade D because of the low number of specimens analysed. The remaining species showed some level of taxonomic unreliability, either for displaying relatively high intraspecific divergences (11.8% of the species, Grade C) or because of conflicting species assignments (3.9% of the species Grade E) (Table 2).

**Table 2:** Attribution of grades (A to E)^1^ to DNA barcodes of 102 marine fish species from Portugal, according to the ranking system proposed in this study.

Species	Grade	Species	Grade
*Acantholabrus palloni*	A	*Leucoraja naevus*	A
*Anthias anthias*	A	*Liza ramado*	A
*Antonogadus megalokynodon*	B	*Lophius budegassa*	A
*Argentina sphyraena*	A	*Macroramphosus scolopax*	A
*Arnoglossus imperialis*	A	*Malacocephalus laevis*	A
*Arnoglossus laterna*	A	*Merluccius merluccius*	A
*Arnoglossus rueppelii*	A	*Microchirus azevia*	A
*Aspitrigla cuculus*	A	*Microchirus boscanion*	B°
*Belone belone*	A°	*Microchirus variegatus*	A
*Benthodesmus simonyi*	A	*Micromesistius poutassou*	A
*Blennius ocellaris*	A°	*Mola mola*	C
*Boops boops*	A	*Molva molva*	A°
*Callionymus lyra*	B°	*Mullus barbatus*	A
*Callionymus maculatus*	D°	*Mullus surmuletus*	A
*Capros aper*	A	*Nezumia sclerorhynchus*	C°
*Centrophorus granulosus*	E	*Pagellus acarne*	A
*Cepola macrophthalma*	C	*Pagellus erythrinus*	C
*Chaunax pictus*	C	*Peristedion cataphractum*	A
*Chelidonichthys lucernus*	A	*Phycis blennoides*	A°
*Chelidonichthys obscurus*	B°	*Platichthys flesus*	A
*Chimaera monstrosa*	A	*Polymetme corythaeola*	E
*Chlorophthalmus agassizi*	A	*Pontinus kuhlii*	D°
*Citharus linguatula*	C	*Raja brachyura*	A
*Coelorinchus caelorhincus*	A°	*Raja clavata*	A
*Conger conger*	A°	*Raja miraletus*	A
*Cyttopsis rosea*	C°	*Raja montagui*	A
*Deania profundorum*	B°	*Sardina pilchardus*	A
*Dicologlossa cuneata*	C	*Scomber colias*	A
*Diplodus annularis*	C	*Scomber scombrus*	A
*Diplodus sargus*	A	*Scorpaena notata*	C
*Dipturus batis*	A	*Scyliorhinus canicula*	A
*Dipturus oxyrinchus*	A	*Serranus cabrilla*	A
*Echiichthys vipera*	A°	*Serranus hepatus*	A
*Engraulis encrasicolus*	A	*Solea senegalensis*	A
*Etmopterus pusillus*	A	*Solea solea*	A
*Etmopterus spinax*	A	*Spicara maena*	C
*Eutrigla gurnardus*	A	*Spondyliosoma cantharus*	A°
*Facciolella oxyrhyncha*	B°	*Symphurus nigrescens*	B°
*Gadiculus argenteus*	A	*Synaphobranchus kaupii*	C
*Gaidropsarus mediterraneus*	A°	*Synchiropus phaeton*	B°
*Galeus atlanticus*	A°	*Torpedo marmorata*	A°
*Galeus melastomus*	A°	*Torpedo nobiliana*	E°
*Halobatrachus didactylus*	D°	*Trachinus draco*	A°
*Helicolenus dactylopterus*	A	*Trachurus picturatus*	A
*Hyperoplus lanceolatus*	A°	*Trachurus trachurus*	A
*Labrus mixtus*	A	*Trachyrinchus scabrus*	E°
*Lepidorhombus boscii*	A	*Trigla lyra*	A
*Lepidorhombus whiffiagonis*	A	*Trigloporus lastoviza*	A
*Lepidotrigla cavillone*	A°	*Trisopterus luscus*	A
*Lepidotrigla dieuzeidei*	A°	*Trisopterus minutus*	A
*Leucoraja circularis*	A	*Xenodermichthys copei*	A

1 Grade A: External Concordance; Grade B: Internal Concordance; Grade C: Sub-optimal Concordance; Grade D: Insufficient Data; Grade E: Discordant Species Assignments. Information is detailed in the text.

°new addition to the global COI-5P library.

The 102 species examined here were initially identified through taxonomic keys, but species level identifications were not possible for 5 additional taxa, which were only assigned to family (Myctophidae, Lotidae, Rajidae, Sternoptychidae) or class (Actinopterygii). We used the BOLD-IDS to putatively assign species names to these specimens. In the first case, samples initially identified as Lotidae, Rajidae and Sternhoptychidae could be putatively assigned to species of the same respective families. In contrast, specimens assigned only to the family Myctophidae and to the class Actinopterygii could not be assigned to species, due to a lack of matching sequences in the database. Since these few specimens were assigned a species name using DNA barcodes, they were excluded from our ranking assessment.

## Discussion

### The fish barcode library and DNA barcode discriminatory ability

Our study confirms the suitability of cytochrome oxidase I (COI-5P) barcodes to identify most fish species within established species boundaries. In fact, specimens morphologically assigned to one species unambiguously clustered within one monophyletic array of sequences, supporting the DNA barcode discriminatory ability and its capacity to provide congruent species-level diagnostics. This study with 659 barcodes for 102 fish species of Portugal represents the start of a reference library for Portuguese fishes. Thirty-three of these species barcodes were new additions to the public library of COI-5P of fish species (Table 1). The dataset aims to contribute to bridge species identification gaps, and to bring additional accuracy to identifications. We verified DNA barcoding as an empirical tool to distinguish closely-related species and we established criteria to rank within and across projects congruencies in species identifications.

The conspecific (0.32%) and congeneric (8.84%) genetic distances observed in this study are within the values usually observed in DNA barcodes studies of fish species (Table 1) [13], [21]. The pattern of COI-5P barcode variation in our regional dataset is broadly concordant with patterns observed in the ichthyiofauna of other regions, e.g. [13], [21]. Although there is some overlap in the distribution of within-species and congeneric distances, the majority of the species showed no overlap (Fig. 3). Exceptions to this trend will be discussed below. Overall, the various taxa also grouped according to their taxonomic hierarchy in monophyletic clusters in the NJ tree. Therefore, the NJ-topology of COI-5P and the established taxonomic hierarchy were largely congruent.

### Ranking system

Most identification and quantification systems rely on the comparison of an unknown sample to a reference specimen. This is also the case in the current and most widespread biological identification system, where a holotype description serves as a standard reference against which unknown specimens are compared for routine identifications [22]. The establishment of a successful identification system based on DNA barcodes depends on the association between an existing taxonomic standard (e.g. a holotype description) and an array of exclusive and closely similar DNA sequences, followed by their integration into dedicated databases – the RLDB [7].

Because biological identification systems are not static, being continuously reviewed and updated in line with changes in alpha taxonomy, DNA barcode libraries must also accommodate protocols for data curation and revision to secure their validity. Similar to whole genome projects, taxonomic databases would greatly benefit from annotation protocols and possibly from ranking systems attributing different levels of reliability to records. Curation and revision is already standard practice in most taxonomic databases, and there are increasing numbers of examples and claims for further refinement of the validation system, namely including annotation (e.g. Encyclopedia of Life; [23]).

The earlier implemented “barcode keyword”, used to highlight DNA barcodes that follow a community standard in one of the major DNA data repositories (International Nucleotide Sequence Database Collaboration (INSDC); [24], [25]), shall not be confused with the ranking system we are proposing here. Although the barcode keyword is a valuable operational asset for data quality control, it does not incorporate any empirically-derived estimate of taxonomic congruence and validity (i.e. concerning the accuracy of the match between a species name and a DNA barcode sequence). Similarly, the confidence levels attributed to original morphology-based identifications (pre-barcode) described in the FISHBOL collaborators' protocol [26] are distinct from the ranking system proposed here, although they could co-exist. Therefore a ranking system located at the end of the analytical chain of the assemblage of reference DNA barcodes (post-barcode ranking), is still required to accommodate operational shortcomings and taxonomic uncertainties. We choose to propose a ranking system that could be readily implemented by any researcher to its own reference library using nothing but the tools currently available in BOLD, and that is flexible enough to accommodate later refinement of the grade attribution criteria. For example, we apply a 2% maximum intra-specific divergence criterion to help defining grades A–C. We know from extensive fish COI-5P datasets that this criterion will apply to the majority but certainly not all fish species [20], or indistinctly to other animal taxa. However, we use this criterion only as a measure of the external, internal or sub-optimal concordance of the taxonomic identifications versus DNA barcodes, within and among reference libraries. Therefore it would be possible to replace the 2% criterion by other metrics deemed more appropriate (e.g. character-based approaches or new prospective algorithms to be implemented in future BOLD versions) as long as the key principles of cross-library concordance are maintained. Below we describe the rationale and features for the ranking system proposed.

- Dynamic management and curation of reference libraries – species discovery, delineation and description is a continuous process that may involve new species discoveries, as well as taxonomic revisions and refinement of pre-existing descriptions and boundaries. A ranking system could help integrate such dynamics in the databases by allowing rank change in line with new evidence.
- Provide flexibility to incorporate uncertainty when required – species delineation hurdles are not equally challenging among taxonomic complexes. For instance, taxa showing moderate levels of within-species divergence, combined with little or no morphological differentiation are particularly problematic. But even in a taxon displaying large divergences species assignments can be ambiguous, especially when data are scarce and taxonomic synthesis of the species complex has not been exhaustive.
- Pursue independent confirmation – DNA barcode libraries have the great advantage of enabling immediate and straightforward comparison of DNA sequences, thus providing a real time congruence check among independent observers and methods. As with any scientific enterprise, the creation of a RLDB can be susceptible to human error. Inaccurate morphology-based identifications, e.g. [10], mislabeling or cross-contamination are among the occasionally diagnosed sources of error that may compromise the validity of DNA barcodes. Hence, external and independent confirmation by finding matching reference barcodes from other researchers would be assigned a higher rank and provide greater confidence in the accuracy of identifications.
- Consider sample size (and geographic breadth) – ideally, DNA barcode records of a given species included in a reference library should comprise multiple specimens collected from representative locations across the known species range. Such sampling schemes may not always be possible due to logistical constraints, at least in the short term. However, if only one or two specimens were collected from a single location, as a precautionary measure, one might consider waiting for collections from elsewhere, or for matching sequences in the database, to attribute a higher rank to those records.

Below we describe examples of our dataset, to illustrate design features and utility of the ranking system for DNA barcode reference records described above. Globally, our ranking system applied to marine fish of Portugal yielded a majority of congruent and externally confirmed DNA barcodes, with circa 73.53% of the species ranking grade A. Species barcodes assigned with grade B (7.84%) await availability of matching sequences, which may shift them to grade A, and ultimately improve the overall rank of the species represented the reference library. Species assigned with grade C (11.76%) will need to be explored with additional molecular markers. These species might either be confirmed as initially described, or might require taxonomic revisions, accordingly to the pattern of divergence revealed by other markers.

From novel evidence that emerged from separate taxonomic studies, we have two examples of species assignments in the dataset that changed since their initial morphological identification. In the initial dataset we had multiple specimens of two species of *Macroramphosus* – *M. scolopax* and *M. gracilis* – whose barcodes showed very little or no divergence, and clustered together. According to our ranking scheme these barcodes were attributed the D grade. Later, a published revision of these taxa [27] concluded that there was no morphological or molecular – at both the mitochondrial control region and the nuclear first S7 intron loci – evidence for the existence of two separate species. Therefore, according to the authors, the two species represent in fact synonyms of a single species, with *M. scolopax* being the valid name. Such new information prompted a review of the specimens and their identifications here, henceforth concluding that all were indeed *M. scolopax*, and concomitantly changed the respective barcode records to grade A.

Among the catsharks of the genus *Galeus*, two species have been recorded in Portuguese continental waters, *G. melastomus* and *G. atlanticus*. Due to their subtle morphological differences, until recently *G. atlanticus* was thought to represent the same species as *G. melastomus*. DNA barcodes from our specimens initially identified as *G. melastomus* were distributed into two distinct clusters diverging on average by 2.7%. The moderate divergence raised uncertainty about the accuracy of the species assignment, and barcodes were ranked grade D. Supported by morphometric and molecular evidence, Castilho et al. (2007) [12] confirmed the occurrence of *G. atlanticus* in Portuguese waters. Because the loci used by Castilho et al. (2007) [12] did not include COI-5P, we were not able to compare them directly with our sequences, but we were provided access to tissue samples of both species [12]. After analyses we could assign unequivocally each barcode cluster of the putative *G. melastomus* to the respective catshark species, which were attributed grade B.

Our dataset includes 7 specimens which were morphologically assigned to the scorpion fish *Scorpaena notata*. These specimens separate into two clear clusters (5 vs 2 specimens) in the NJ tree, diverging on average by 18.5%. Such a high level of divergence is at the extreme top range of COI-5P congeneric divergences usually observed in marine fish [20], and well within average divergences between members of a family. Thus, the two clusters likely represent 2 distinct species (Grade C). The search for homologous sequences in BOLD was not conclusive. Specimens from each cluster both matched *S. notata* barcodes from other projects available from BOLD. The possibility that sister species of *Scorpaena* were confused with *S. notata* was not confirmed. None of our specimens of *S. notata* matched any of the two other sister species of *Scorpaena*, namely *S. elongata* and *S. scrofa*. This group of 3 named species of *Scorpaena*, separates into 4 distinct and fairly divergent clades, suggesting, solely from a molecular perspective, the presence of 4 species. However, *S. elongata* and *S. scrofa* are not completely separated into their respective clades, overlapping within each clade (together with a few *S. notata* from other projects).

Because barcodes appear to resolve the scorpion fish species complex into separate clades, we attributed mismatches to inaccurate identifications rather than to incomplete lineage sorting. Indeed, this is not so surprising, as scorpion fish are often notoriously difficult to discriminate morphologically [13]. Although alternative hypothesis such as introgressive hybridization or paraphyly cannot be discarded, in this case, molecular data appears to be one step ahead of currently described morphological variation. If a ranking system for the DNA barcode libraries were already implemented, it would have helped to pinpoint BOLD records of *Scorpaena* spp. with higher level of reliability, while the uncertain status of other would become more evident. Detailed taxonomic inspection and eventual revision could then be prioritized towards the uncertain records.

### Concluding remarks

Here we present a RLDB for the first hundred fish species of Portugal, and illustrate the utility of the reference database and of a grading system for categorizing taxonomic reliability. The pattern of variation of the COI-5P barcodes concurred with patterns observed previously for marine fish barcode libraries, with average within species divergence being substantially smaller then congeneric divergences. Aside from a few exceptions, species assignments were generally straightforward, with species barcodes forming unambiguous monophyletic clusters.

Although these features provide a prime indication of the reliability of the reference library, there is no established approach to verify cross-library taxonomic congruence or global validation of a DNA barcode-species name match. An independent validation (peer-library validation) is critical for the success of a truly global and accurate species identification system, given the dynamic nature of taxonomic discovery, and the inherent complexity and source of variation in any such classification system.

We thereby propose a ranking system for the RLDB that incorporates such cross-validation. This framework would allow for continuous refinement in line with increasing availability of public data and the iterative nature of taxonomic evidence. By doing so we augment the utility of our case-study library of marine fish of Portugal, providing enhanced accuracy to potential end-users, while elucidating taxonomic relationships presented in comparable data sets. However, the realised utility of a ranking system such as the one proposed can become effective only if globally implemented. Regardless of the ultimate structure of any such comparative and standardised ranking system, its availability would be expected to extend the utility and accessibility of DNA barcode reference databases across a diverse end-user community.

## Supporting Information

Figure S1 **Neighbour Joining tree of fishes of Portugal.** NJ Tree resulting from 659 sequences and obtained using Kimura-2-parameter distance model. Branches are collapsed at species level and supported by bootstrap values based on 1000 replicates. In total, 102 species, 79 genera, and 54 families are here reported.
(TIF)

Table S1 **Accession numbers for DNA barcodes used in this study.** Specimens' list of 659 COI-5P sequences from 102 species, 79 genera, 54 families of marine fishes from Portugal. The corresponding BOLD – Process IDs and Project names are provided.
(XLS)

Table S2 **Succession of species used to generate the Klee diagram.**
(XLS)
